# Biochar-Stabilized Tea Tree Oil in Chitosan Membranes for Sustainable Antimicrobial Packaging

**DOI:** 10.3390/molecules31071079

**Published:** 2026-03-25

**Authors:** Kang Zhang, Jing Sun, Peiqin Cao, Yixuan He, Yixiu Wang, Hongxu Zhu

**Affiliations:** 1 Department of Chemistry and Chemical Engineering, Hunan Institute of Science and Technology, Yueyang 414006, China; 12021019@hnist.edu.cn (K.Z.); 14192003540@vip.hnist.edu.cn (J.S.); 12021028@hnist.edu.cn (P.C.); 2 Qingyun Zhixing Technology Co., Ltd., Yueyang 414006, China; 3 Department of Hepatic Surgery, Fudan University Shanghai Cancer Center, Shanghai 200032, China; yixiuwang@fudan.edu.cn

**Keywords:** lotus stalk biochar, controlled-release active packaging, tea tree oil encapsulation, chitosan-based composite membrane, multifunctional food preservation

## Abstract

This study developed an active packaging material by incorporating tea tree oil (TTO)-loaded lotus stalk biochar (BC@TTO) into a chitosan (CS) matrix. Biochar was prepared from lotus stalks via pyrolysis at 600 °C and characterized, revealing a mesoporous structure with a specific surface area of 35.9 m^2^/g. Adsorption studies demonstrated that BC exhibited high affinity for TTO, following pseudo-first-order kinetics and the Langmuir isotherm model, with a maximum adsorption capacity of 295.6 mg/g. Chitosan-based composite membranes with varying BC@TTO contents (1–7 wt%) were fabricated by solution casting. The incorporation of BC@TTO significantly enhanced the tensile strength, elongation at break, barrier properties (water vapor and oxygen), and antioxidant/antibacterial activities of the membranes, with optimal performance observed at 3 wt% loading. However, higher loadings led to filler aggregation, reduced transparency, and compromised mechanical properties. In vitro release studies indicated that TTO release followed the Avrami model, suggesting a diffusion-controlled mechanism. Preservation tests on blueberries showed that the CS-3BC@TTO membrane effectively reduced weight loss and maintained fruit quality during storage. This work presents a promising strategy for designing bioactive packaging materials with sustained release functionality for food preservation applications.

## 1. Introduction

Active food packaging has emerged as a strategic approach to extend shelf life and maintain food quality by incorporating functional agents into the packaging matrix. Chitosan (CS), a biodegradable and antimicrobial biopolymer, is a promising base material for such applications [1,2]. However, its inherent limitations, including poor barrier properties against moisture and gases, moderate mechanical strength, and the often-rapid, uncontrolled release of active compounds, hinder its practical performance [3]. To address these challenges, the integration of nanofillers as both reinforcing agents and carriers for bioactive substances presents a viable solution [4,5,6,7]. Biochar (BC), a carbon-rich porous material derived from biomass pyrolysis, offers exceptional adsorption capacity, stability, and compatibility with biopolymers [8,9,10,11]. Its application as a dual-functional filler—simultaneously enhancing polymer properties and acting as a sustained-release reservoir for active ingredients—remains underexplored in packaging science, particularly when derived from unconventional agricultural waste [12].

Tea tree oil (TTO) is a potent natural antimicrobial and antioxidant agent to confer bioactivity to packaging membranes [13,14]. While TTO offers a green alternative to synthetic preservatives, its direct incorporation into polymer matrices is hampered by high volatility, susceptibility to oxidation, and potential negative impacts on the membrane’s mechanical integrity due to its plasticizing effect [15]. To overcome these drawbacks, we propose a novel stabilization and delivery strategy using BC. Biochar, a carbon-rich porous material derived from the pyrolysis of lotus stalks—an abundant agricultural waste, serves as an ideal host [16]. Its high surface area and adsorption capacity are hypothesized to effectively encapsulate and protect TTO, mitigating its volatility and enabling sustained release. This design transforms TTO from a liability into a stable, functional component, while the BC itself acts as a reinforcing nanofiller aimed at improving the composite’s physical properties.

The central scientific hypothesis of this work is that the micro/mesoporous structure of lotus stalk biochar will facilitate high-capacity, stable loading of TTO via monolayer adsorption, and that the subsequent incorporation of this BC@TTO complex into a chitosan matrix will yield a composite membrane with significantly enhanced mechanical strength, improved water vapor and oxygen barrier properties, and sustained release kinetics of TTO. This sustained release is expected to confer prolonged, dose-dependent antioxidant and antibacterial activities, ultimately leading to superior preservation efficacy for perishable fruits. To test this hypothesis, this research systematically investigates (1) the structural characteristics and adsorption behavior of BC for TTO, (2) the physicochemical, mechanical, barrier, and functional properties of the CS/BC@TTO composite membranes, and (3) the release kinetics of TTO and the practical preservation performance of the optimized membrane on blueberries.

## 2. Results

### 2.1. Structure Analysis of BC

**Figure 1:**
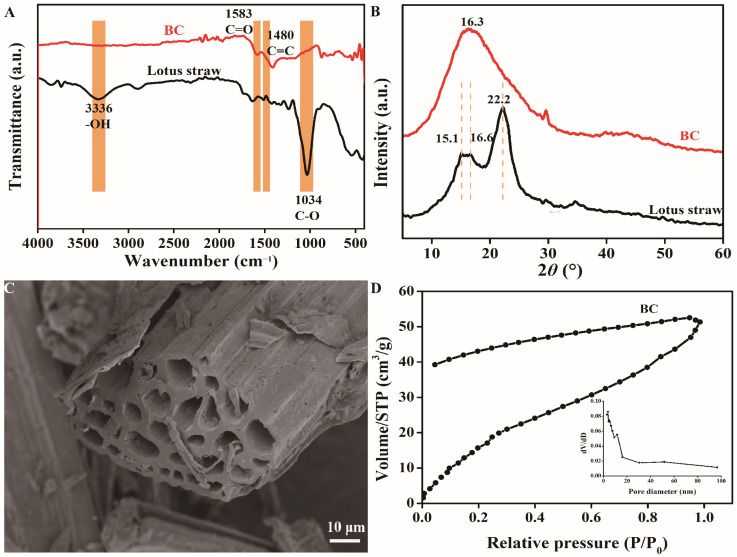
Structural characterization of lotus straw and BC, (**A**) FTIR, (**B**) XRD, (**C**) SEM, and (**D**) BET.

The FTIR spectra of lotus straw and BC are shown in Figure 1A. The broad band around 3336 cm^−1^ and the strong band around 1034 cm^−1^ in the lotus straw spectrum are attributed to O–H stretching vibrations and C–O stretching vibrations in polysaccharides, respectively. The peaks at 1583 cm^−1^ and 1480 cm^−1^ in the BC spectrum are associated with C=O and aromatic C=C stretching vibrations, respectively [17]. The reduction in O–H and C–O bands confirms the decomposition of cellulose, hemicellulose, and other oxygenated functional groups, leading to a more carbonaceous and aromatic structure. The increase in C=C band intensities suggests the development of condensed aromatic rings and stable oxygen-containing moieties [18]. These structural changes are consistent with enhanced thermal stability, reduced hydrophilicity, and potential applicability of lotus straw-derived biochar in environmental adsorption, carbon sequestration, or soil amendment.

XRD patterns of lotus straw and BC are shown in Figure 1B. Two peaks at 16.0° and 22.2° in the XRD pattern of lotus straw correspond to the (101) and (002) planes of cellulose I, indicating a microcrystalline structure with partial crystalline order [19]. In contrast, the biochar pattern shows a very broad and diffuse hump. The disappearance of sharp crystalline peaks and the emergence of a broad feature indicate the conversion of the ordered biomass structure into a largely amorphous carbon matrix during pyrolysis [20]. The amorphous and turbostratic structure of biochar contributes to its high porosity and surface area, which provide abundant adsorption sites for TTO, while its chemical stability ensures that the biochar remains inert and does not exhibit high reactivity. This makes it ideal for stabilizing and adsorbing TTO for sustained release applications

SEM image of the BC is shown in Figure 1C. The BC exhibits a rough and heterogeneous texture with visible pores and crevices, indicative of the development of a porous carbon matrix following pyrolysis. This morphology is typical of biomass-derived biochars, resulting from the thermal decomposition and volatilization of organic components [21].

The N_2_ adsorption–desorption curves are shown in Figure 1D. The derived specific surface area is 35.9 m^2^/g, and the average pore diameter is 3.4 nm, classifying the predominant pore structure within the mesoporous range. The combination of this accessible surface area and mesoporous architecture suggests that the lotus straw biochar has suitable physicochemical properties for use as an adsorbent.

### 2.2. The Adsorption Behavior of BC to TTO

**Figure 2:**
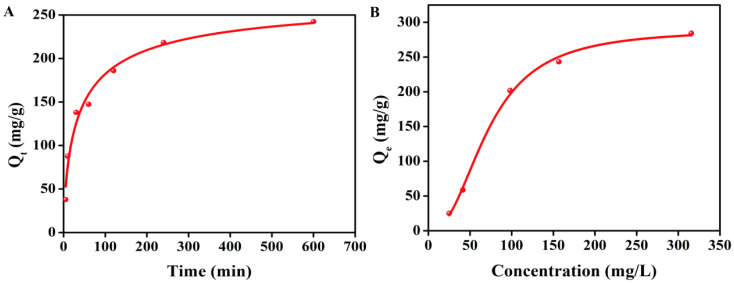
The adsorption behavior of BC to TTO, (**A**) Adsorption kinetics, and (**B**) Adsorption isotherm.

The adsorption kinetics of TTO onto BC are shown in Figure 2A. The adsorption proceeds rapidly in the initial stage due to the abundant active sites on BC and the high concentration gradient of TTO. The rate gradually decreases over time, consistent with the exponential trend of the model. The process follows the pseudo-first-order kinetic model, with the fitted equation ln(242.5 − *Q*_t_) = ln242.5 − 4.1 × 10^−2^ × *t* and the correlation coefficient R^2^ = 0.97. The theoretical equilibrium adsorption capacity is 242.5 mg/g, which is slightly higher than the experimentally observed final adsorption capacity (235.0 mg/g). This indicates that the model reliably predicts the long-term adsorption trend while also suggesting that complete equilibrium was not fully reached within the experimental timeframe. The high R^2^ value suggests that the pseudo-first-order model adequately describes the adsorption process. Pseudo-first-order kinetics is often associated with physisorption or diffusion-controlled processes, suggesting that the adsorption of TTO onto BC may be dominated by physical interactions such as van der Waals forces or pore filling [22].

The adsorption isotherm of TTO onto BC is presented in Figure 2B. The experimental data were well fitted by the Langmuir isotherm model, with the linearized equation *C*_e_/*Q*_e_ = *C*_e_/295.6 + 1.0 and the correlation coefficient R^2^ = 0.98. The high correlation coefficient confirms that the Langmuir model provides a good description of the adsorption equilibrium. The strong fit to the Langmuir isotherm suggests that TTO adsorption onto BC occurs primarily via monolayer coverage on a homogeneous surface with a finite number of identical adsorption sites [23]. The calculated maximum adsorption capacity of 295.6 mg/g reflects a relatively high adsorption capability of BC for TTO under the studied conditions, consistent with the kinetic results showing rapid and substantial uptake. The well-defined monolayer saturation capacity provides a useful parameter for designing and scaling up adsorption systems for TTO removal or recovery.

### 2.3. Structure Analysis of Chitosan-Based Active Packaging Membranes

**Figure 3:**
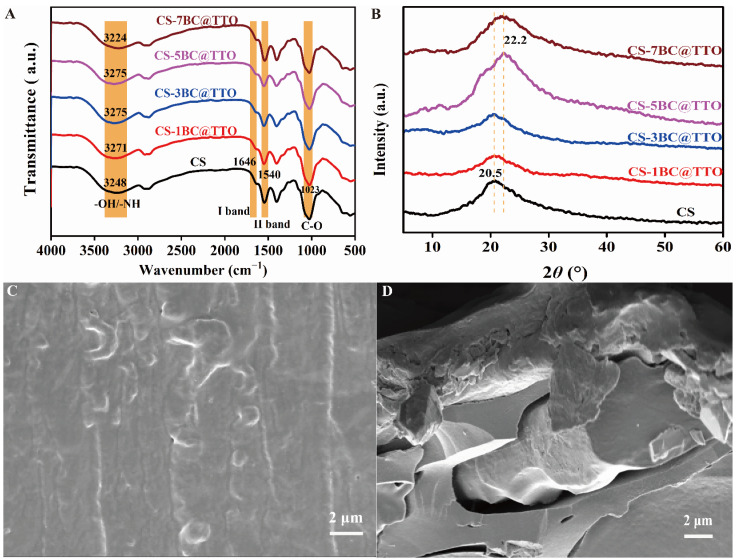
Structural characterization of chitosan-based active packaging membranes, (**A**) FTIR, (**B**) XRD, (**C**) SEM image of CS-3BC@TTO, and (**D**) SEM image of CS-7BC@TTO.

The FTIR spectra of chitosan-based active packaging membranes with BC@TTO are presented in Figure 3A. All samples exhibit strong and broad absorption bands within the 3224–3275 cm^−1^ region, which are attributed to the overlapping O–H stretching vibrations and N–H stretching vibrations characteristic of chitosan. Peaks at 1646 cm^−1^ and 1540 cm^−1^ correspond to amide I (C=O stretching) and amide II (N–H bending/C–N stretching), respectively [24]. The peak at 1023 cm^−1^ is assigned to C–O stretching vibrations associated with the polysaccharide backbone and primary alcohol groups. As shown in the provided data, the O–H/N–H stretching region shows slight variations in peak positions among different samples, which may be related to changes in hydrogen bonding interactions due to the incorporation of BC and TTO [25]. Such interactions could enhance the compatibility and stability of the composite membrane.

XRD patterns of chitosan-based active packaging membranes incorporated with different amounts of BC@TTO are presented in Figure 3B. All samples exhibit a broad diffraction peak centered at approximately 20.5° (2θ), which corresponds to the characteristic crystalline reflection of chitosan, specifically associated with the (110)/(200) lattice planes [26]. This confirms that the chitosan matrix maintains its semicrystalline structure in the composite membranes. The crystallinity of the membranes was calculated by Jade 6.0 based on the XRD data. The results show that the crystallinity varies with the composition, with calculated values of 23.6% (CS), 29.5% (CS-1BC@TTO), 31.1% (CS-3BC@TTO), 26.5% (CS-5BC@TTO), and 24.5% (CS-7BC@TTO) for the different composite membranes. The initial increase in crystallinity suggests that BC@TTO particles may act as nucleation sites, promoting the ordered arrangement of chitosan chains and enhancing crystal formation [27]. However, at higher BC@TTO concentrations, the crystallinity decreases. This can be attributed to the disruptive effect of excessive filler content, which may hinder the close packing and hydrogen bonding of chitosan chains, leading to greater structural disorder [28]. This optimized structural change, particularly the peak crystallinity observed at intermediate BC@TTO content, likely contributes to an improved balance between mechanical integrity and functional performance, making these composites promising for active packaging applications.

The surface morphologies of chitosan-based composite membranes loaded with different amounts of BC@TTO were investigated using SEM. Representative images of CS/3BC@TTO and CS/7BC@TTO are shown in Figure 3C and Figure 3D, respectively. The surface of CS/3BC@TTO appears relatively smooth and homogeneous at this magnification, indicating good compatibility and integration between the filler and the polymer. At a lower loading (3 wt%), the particles can be effectively wetted and separated by the chitosan solution during membrane formation, leading to a well-dispersed composite [29]. However, the surface morphology of CS/7BC@TTO becomes rougher and more heterogeneous, with visible aggregates distributed unevenly across the membrane. At a higher loading (7 wt%), the increased particle density promotes filler-filler interactions (e.g., van der Waals forces), overcoming the stabilizing effect of the polymer matrix and resulting in agglomeration [30]. This morphological difference is a critical factor explaining the variations in physicochemical and functional properties among the composites, underscoring the importance of optimizing filler loading to achieve a homogeneous, high-performance active packaging material.

### 2.4. Transparency of Chitosan-Based Active Packaging Membrane

**Figure 4:**
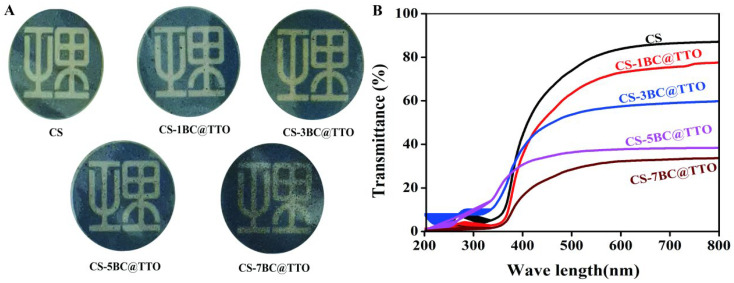
Transparency of chitosan-based active packaging membranes, (**A**) Photos, and (**B**) Transmittance.

The optical characteristics of chitosan-based active packaging membranes with varying BC@TTO content are presented in Figure 4. In Figure 4A, the pure CS membrane is highly transparent and colorless. With the addition of BC@TTO, the membranes become progressively less transparent and darker. The CS-7BC@TTO membrane appears nearly opaque, indicating significant light scattering or absorption by the incorporated filler. In Figure 4B, the pure CS membrane shows high transmittance across the measured wavelength range, which is consistent with its high transparency. The incorporation of BC@TTO into chitosan membranes significantly enhances their UV-barrier properties but reduces their transparency in the visible range. The degree of transparency loss correlates with filler content and its dispersion state, with the CS-7BC@TTO composite becoming essentially opaque. This tunable optical property, combined with the active functionality, allows for the design of packaging materials tailored to protect specific light-sensitive food products, where the excellent UV blocking can help extend shelf life despite the reduction in visual clarity [31].

### 2.5. Tensile Properties of Chitosan-Based Active Packaging Membranes

**Figure 5:**
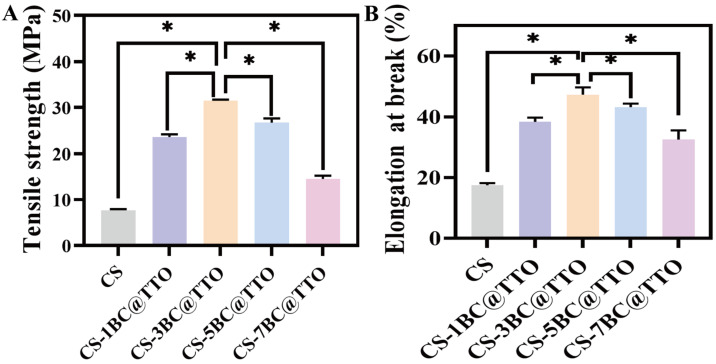
(**A**) Tensile strength and (**B**) elongation at break of chitosan-based active packaging membranes. Asterisks (*) indicate *p* < 0.05.

The tensile strength and elongation at break of chitosan-based active packaging membranes are shown in Figure 5. The pure CS membrane exhibited a tensile strength of 21.1 ± 1.3 MPa and an elongation at break of 23.3 ± 0.1%. Upon incorporation of BC@TTO, both mechanical properties initially improved, then declined as the BC@TTO content increased. At 3% BC@TTO loading, the tensile strength reached a maximum of 31.7 MPa, representing a significant increase of approximately 61.0% compared to the pure CS membrane (*p* < 0.05). Similarly, the elongation at break peaked at 45.7%, corresponding to a 94.0% improvement, which was also statistically significant (*p* < 0.05). However, further increasing the BC@TTO content to 5% and 7% led to a noticeable reduction in both tensile strength and elongation at break. This decline is attributed to filler agglomeration beyond a critical concentration, which introduces stress concentration points and structural defects, thereby compromising the homogeneity of the composite matrix and weakening its mechanical performance [32]. The significant enhancements observed at optimal BC@TTO loading suggest that these composite membranes possess suitable mechanical properties for potential packaging applications.

### 2.6. Moisture Absorption and Gas Barrier

**Figure 6:**
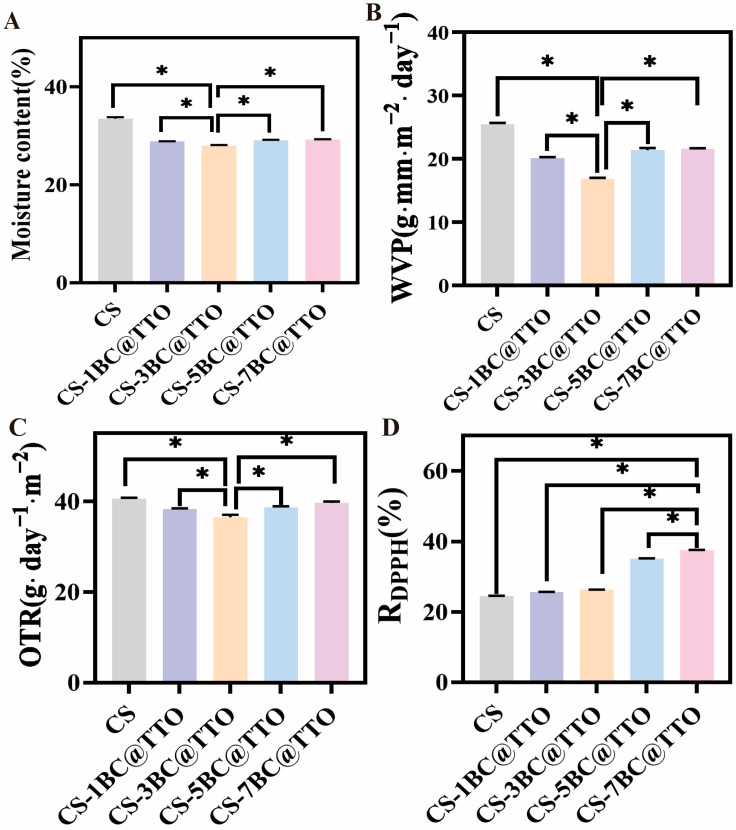
(**A**) Moisture contents, (**B**) water vapor permeability, (**C**) oxygen transmission rate, and (**D**) DPPH free radical scavenging rate of chitosan-based active packaging membranes. Asterisks (*) indicate *p* < 0.05.

The moisture absorption properties of the chitosan-based composite membranes are presented in Figure 6A. The pure CS membrane exhibited a moisture absorption content of 33.3 ± 0.6%. With the incorporation of BC@TTO, the moisture absorption showed an overall decreasing trend. Specifically, the CS/1BC@TTO, CS/3BC@TTO, CS/5BC@TTO, and CS/7BC@TTO composites recorded moisture absorption values of 28.9 ± 0.1%, 25.7 ± 1.4%, 29.1 ± 0.2%, and 29.2 ± 0.1%, respectively. The lowest absorption was observed for CS/3BC@TTO, which decreased by approximately 22.8% compared to the pure CS membrane, a statistically significant reduction (*p* < 0.05). The decline in moisture absorption upon the addition of BC@TTO can be attributed to the hydrophobic nature of the incorporated filler, which reduces the number of hydrophilic sites available for water interaction within the composite matrix. The significant reduction observed at lower BC@TTO loadings (1% and 3%) indicates effective dispersion and interaction between BC@TTO and the chitosan matrix, thereby enhancing the composite’s moisture resistance. However, the slight increase in moisture absorption at higher BC@TTO contents (5% and 7%) suggests possible filler agglomeration, which may create localized defects or interfacial gaps that facilitate moisture uptake [33]. These results demonstrate that an optimal BC@TTO content (around 3%) can effectively improve the hydrophobicity of chitosan-based membranes, which is desirable for packaging applications where moisture barrier properties are critical.

WVP of the chitosan-based composite membranes is shown in Figure 6B. The pure CS membrane displayed a WVP of 25.4 ± 0.4 g·mm·m^−2^·day^−1^. With the addition of BC@TTO, the WVP decreased significantly at lower loadings. Specifically, the WVP values for CS/1BC@TTO, CS/3BC@TTO, CS/5BC@TTO, and CS/7BC@TTO were 20.2 ± 0.3, 17.0 ± 1.0, 21.6 ± 0.4, and 21.7 ± 0.9 g·mm·m^−2^·day^−1^, respectively. The minimum WVP was observed for CS/3BC@TTO, which was approximately 33.1% lower than that of the pure CS membrane, a reduction that was statistically significant (*p* < 0.05). The notable decrease in WVP with the incorporation of BC@TTO can be attributed to the hydrophobic nature of BC@TTO, which enhances the tortuosity of the diffusion path for water vapor through the composite matrix [34]. The significant reduction at 3% BC@TTO suggests that the filler is well dispersed at this concentration, forming an effective barrier network that impedes moisture transmission. The subsequent increase in WVP at higher BC@TTO loadings (5% and 7%) may result from filler aggregation, which could create micro-gaps or interfacial defects that facilitate vapor permeation. These findings indicate that an optimal BC@TTO content (around 3%) substantially improves the water vapor barrier properties of chitosan membranes, which is advantageous for packaging applications requiring controlled moisture exchange.

OTR of the chitosan-based composite membranes is presented in Figure 6C. The pure CS membrane exhibited an OTR of 40.4 ± 1.8 g·m^−2^·day^−1^. The incorporation of BC@TTO led to a general decrease in OTR at lower loadings. Specifically, the OTR values for the CS/1BC@TTO, CS/3BC@TTO, CS/5BC@TTO, and CS/7BC@TTO composites were 38.4 ± 0.9, 36.1 ± 1.7, 38.9 ± 1.0, and 39.4 ± 2.1 g·m^−2^·day^−1^, respectively. The lowest OTR was achieved by the CS/3BC@TTO membrane, showing a reduction of approximately 10.6% compared to the pure CS membrane, which was statistically significant (*p* < 0.05). The observed reduction in OTR with the addition of BC@TTO is likely due to the increased tortuosity of the diffusion pathway for oxygen molecules within the composite matrix, as well as the potential barrier effect provided by the well-dispersed filler [35]. The significant improvement at 3% BC@TTO indicates that at this optimal loading, the filler creates an effective network that hinders oxygen permeation. The subsequent rise in OTR at higher BC@TTO contents (5% and 7%) may be attributed to filler aggregation, which can induce microstructural defects or interfacial voids, thereby facilitating oxygen transmission. These results demonstrate that a moderate BC@TTO content can enhance the oxygen barrier properties of chitosan membranes, which is beneficial for extending the shelf life of oxygen-sensitive packaged products.

### 2.7. Antioxidant and Antibacterial Activities

R_DPPH_ of the chitosan-based composite membranes is shown in Figure 6D. The pure CS membrane exhibited an R_DPPH_ of 24.6 ± 0.8%. Incorporation of BC@TTO resulted in a marked and concentration-dependent enhancement in antioxidant activity [36]. The R_DPPH_ values for CS/1BC@TTO, CS/3BC@TTO, CS/5BC@TTO, and CS/7BC@TTO were 25.6 ± 0.4%, 26.3 ± 0.7%, 35.1 ± 1.3%, and 37.5 ± 1.1%, respectively. Notably, the scavenging activity significantly increased at BC@TTO loadings of 5% and 7%, with improvements of approximately 42.7% and 52.4% over the pure CS membrane (*p* < 0.05). The progressive enhancement in DPPH radical scavenging capacity is directly attributable to the inclusion of TTO within the composite. TTO is known to contain bioactive compounds such as terpinene-4-ol, which possess potent antioxidant properties [37]. As the BC@TTO content increases, a greater quantity of these active compounds is introduced into the chitosan matrix, leading to a corresponding rise in antioxidant efficacy. This dose-dependent improvement suggests that the antioxidant components within TTO remain accessible and active within the composite structure. The significant boost in radical scavenging activity, especially at higher filler loadings, indicates that these composite membranes could effectively protect packaged contents from oxidative degradation, thereby extending product shelf life.

**Figure 7:**
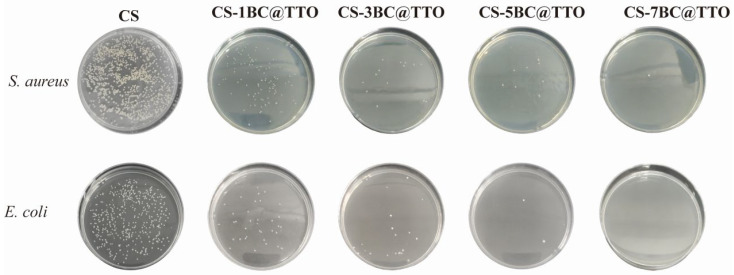
Antibacterial activities of chitosan-based active packaging membranes.

Antibacterial activities of chitosan-based active packaging membranes are shown in Figure 7. Compared to the pure CS membrane, all BC@TTO-incorporated composites exhibited strong and dose-dependent antibacterial effects. The inhibition rates against *E. coli* for CS/1BC@TTO, CS/3BC@TTO, CS/5BC@TTO, and CS/7BC@TTO, as determined by the plate count method, were 88.9%, 95.3%, 98.9%, and 99.9%, respectively. Similarly, the inhibition rates against *S. aureus* were 89.8%, 98.3%, 98.9%, and 99.9% [38]. The antibacterial efficacy showed a significant and progressive enhancement with increasing BC@TTO content, reaching near-complete inhibition (99.9%) at the highest loading of 7% for both bacterial strains. This remarkable antibacterial performance is primarily attributed to the bioactive components of TTO encapsulated within the BC network. Key compounds such as terpinen-4-ol in TTO are known to disrupt microbial cell membranes, leading to leakage of cellular contents and eventual cell death [39]. The dose-dependent increase in inhibition rates confirms the effective release and bioavailability of these antimicrobial agents from the composite matrix. The near-total inhibition achieved at higher BC@TTO loadings (5% and 7%) indicates that the composite membranes can provide a robust barrier against both Gram-negative (*E. coli*) and Gram-positive (*S. aureus*) bacteria. These results suggest that the developed membranes possess strong potential for use in active food packaging, where suppressing microbial growth is critical for extending shelf life and ensuring food safety.

### 2.8. The Release Behavior of Tea Tree Oil in Chitosan-Based Active Packaging Membranes

**Figure 8:**
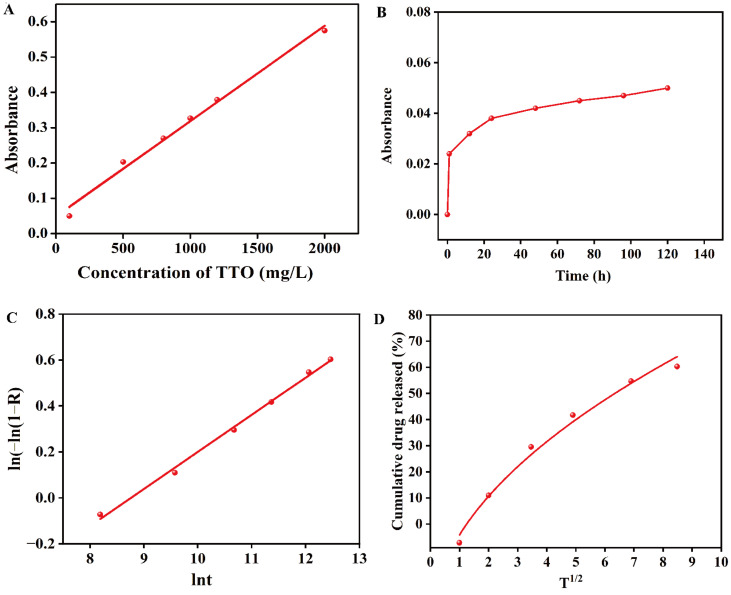
(**A**) Standard curve, (**B**) release kinetics, (**C**) Avrami model, and (**D**) Higuchi model.

The release behavior of TTO from chitosan-based composite membranes was quantitatively evaluated. A standard calibration curve was first established for TTO solution as shown in Figure 8A, showing a strong linear relationship between concentration and absorbance, represented by the equation A = 3 × 10^−4^ × c + 4.9 × 10^−2^ with a high coefficient of determination (R^2^ = 0.999). The cumulative release profiles of TTO from the membranes were subsequently analyzed, as shown in Figure 8B, which illustrates the release kinetics over time. To provide a more comprehensive understanding of the release mechanism, the experimental data were fitted using both the Avrami model (Figure 8C) and the Higuchi model (Figure 8D) for comparison. The Avrami model yielded the equation ln(−ln(1 − R)) = 0.16 × ln t − 1.41, with an excellent fit (R^2^ = 0.996), whereas the Higuchi model, while providing a reasonable approximation, showed a slightly lower correlation coefficient. This comparison indicates that the release is primarily governed by Fickian diffusion through the chitosan matrix, but also affected by polymer relaxation, which is better captured by the Avrami model. The Avrami exponent (*n*) of 0.16, significantly below 1, further supports this diffusion-controlled, sustained release mechanism. This controlled and prolonged release profile is advantageous for active packaging applications, as it implies a gradual and sustained delivery of the antimicrobial agent over time, effectively extending the functional lifespan of the packaging material.

### 2.9. Preservation of Blueberries by Chitosan-Based Active Packaging Membrane

**Figure 9:**
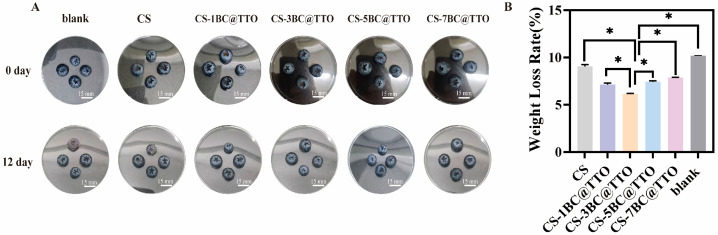
The morphology (**A**) and weight loss rate (**B**) of blueberries preserved for 12 days using chitosan-based active packaging membrane. Data are presented as mean ± SD. Asterisks (*) indicate *p* < 0.05.

Figure 9 illustrates the effect of chitosan-based active packaging membranes on the preservation of blueberries. The blueberries were packaged using the chitosan-based membranes, which formed a protective barrier around the fruit, significantly reducing weight loss and maintaining morphological integrity during storage. After 12 d of storage, blueberries in the control group exhibited noticeable shrinkage, with a weight loss rate reaching 10.17%. Blueberries packaged with the chitosan-based membranes maintained superior morphological integrity compared to the control. The weight loss value of the blueberries packaged using the CS membrane without the addition of TTO was 8.9%. After incorporation of TTO, the weight loss rates were significantly reduced to 7.0% (CS-1BC@TTO), 6.2% (CS-3BC@TTO), 7.4% (CS-5BC@TTO), and 7.9% (CS-7BC@TTO). Statistical analysis indicated that all membrane-treated groups exhibited a significantly lower weight loss rate than the blank control group (*p* < 0.05). Notably, the CS-3BC@TTO membrane demonstrated the most effective moisture retention, with its weight loss rate being significantly lower than that of the pure CS membrane and the groups with higher BC@TTO loadings (5% and 7%) (*p* < 0.05). These results demonstrate that the chitosan-based membrane effectively inhibits moisture evaporation. The preservation efficacy initially improved and then diminished with increasing BC@TTO concentration, exhibiting a non-linear dose–response relationship. This trend is attributed to the formation of a dense, protective barrier on the fruit surface by the composite membrane, which reduces water vapor transmission, transpiration, and respiratory metabolism [40]. However, at excessive BC@TTO loadings (e.g., 5% and 7%), nanoparticle aggregation likely occurs due to van der Waals forces. This aggregation can create stress concentration points and lead to microstructural defects or cracks during membrane formation/drying, forming pathways for moisture diffusion and thereby increasing the weight loss rate again. In conclusion, the chitosan-based active packaging membranes, particularly at an optimal BC@TTO content (3%), significantly prolong the shelf life of blueberries by effectively mitigating post-harvest water loss.

## 3. Discussion

The present study successfully developed a chitosan-based composite membrane functionalized with BC@TTO for active food packaging applications. The findings align with and extend the initial hypothesis that the porous structure of biochar could serve as an effective host for TTO encapsulation, while its incorporation into chitosan would enhance the functional and barrier properties of the resulting composite. The results demonstrate that the BC@TTO filler not only acts as a reinforcing agent but also enables the sustained release of bioactive TTO, leading to improved preservation performance.

The adsorption behavior of BC toward TTO followed pseudo-first-order kinetics and the Langmuir isotherm, indicating a monolayer, diffusion-controlled physisorption process. This is consistent with previous reports on the adsorption of essential oils onto porous carbons, where van der Waals interactions and pore filling dominate. The maximum adsorption capacity of 295.6 mg/g highlights the efficiency of lotus stalk-derived biochar as a carrier for volatile active compounds, supporting its role in stabilizing TTO and mitigating issues such as rapid evaporation and oxidation.

The incorporation of BC@TTO into the chitosan matrix led to significant improvements in tensile strength, elongation at break, and barrier properties against water vapor and oxygen at an optimal loading of 3 wt%. These enhancements can be attributed to the good dispersion of BC@TTO at lower concentrations, which promotes polymer–filler interactions, increases crystallinity, and extends the diffusion pathway for gases and moisture. However, at higher loadings (5–7 wt%), filler aggregation occurred, leading to structural defects and a decline in mechanical and barrier performance. This observation is in agreement with composite theory, where excessive filler content often results in phase separation and compromised matrix integrity.

The sustained release of TTO, well-described by the Avrami model with a low diffusional exponent (*n* = 0.16), confirms a Fickian diffusion-controlled mechanism. This slow and prolonged release is advantageous for active packaging, as it ensures long-term antimicrobial and antioxidant activity without an initial burst effect. The corresponding enhancement in DPPH radical scavenging and antibacterial efficacy against *E. coli* and *S. aureus* further validates the functional viability of the membrane. These results align with studies on essential oil-loaded composites, where controlled release is key to maintaining bioactive performance over time.

In practical preservation tests on blueberries, the CS-3BC@TTO membrane significantly reduced weight loss and maintained fruit quality during storage. This can be attributed to the combined effects of improved moisture barrier properties and the sustained release of TTO, which likely suppressed microbial growth and oxidative spoilage. The non-linear dose–response relationship, with optimal performance at 3% loading, underscores the importance of balancing filler content to avoid aggregation while maximizing functional benefits.

This work contributes to the growing field of sustainable and active packaging by valorizing agricultural waste (lotus stalks) as a source of functional biochar and employing a natural antimicrobial (TTO) as a green preservative. The dual-functionality of BC@TTO—as both a reinforcing filler and a reservoir for active release—offers a versatile strategy for designing next-generation packaging materials. From an environmental perspective, the use of biodegradable chitosan and waste-derived biochar aligns with circular economy principles and reduces reliance on synthetic additives.

However, the reduction in membrane transparency at higher BC@TTO loadings may limit visual appeal in certain packaging applications. This trade-off between functionality and aesthetics is a common challenge in active packaging development and may be addressed in future designs through multilayer structures or surface-functionalized fillers.

To advance this promising system, several research avenues are suggested: Investigating other biomass sources for biochar production to optimize porosity, surface chemistry, and compatibility with different active compounds. Exploring alternative biopolymer matrices or hybrid systems to improve optical clarity without compromising active performance. Evaluating the long-term stability, migration behavior, and safety of BC@TTO composites under real food storage conditions. Extending application tests to other perishable foods (e.g., meats, leafy vegetables) to assess broader efficacy. Incorporating intelligent indicators (e.g., pH-sensitive dyes) to develop “smart” packaging that monitors food freshness in real time.

In conclusion, this study provides a feasible and eco-friendly approach to developing sustained-release active packaging with enhanced preservation performance. By integrating biochar-mediated encapsulation with a biodegradable polymer matrix, this work opens new pathways for functional material design in food packaging and beyond.

## 4. Materials and Methods

### 4.1. Materials and Chemicals

Analytical grade chitosan (CS, C1516215-100g), anhydrous ethanol, calcium oxide (CaO), glycerol, tea tree oil (TTO), ethyl acetate, ascorbic acid, and 2,2-diphenyl-1-picrylhydrazyl (DPPH) were analytical grade, and purchased from Titan Scientific Co., Ltd. (Shanghai, China). The viscous average molecular weight of chitosan was 5.4 × 10^5^ g·mol^−1^. Lotus stalks were purchased from Kangyuan Co., Ltd. (Yueyang, China).

### 4.2. Preparation of Biochar (BC) and TTO Loaded in Biochar (BC@TTO)

Biochar was prepared using lotus stalks as the feedstock. The stalks were first subjected to pretreatment including washing with deionized water, soaking at room temperature, drying, grinding, and sieving. The pretreated material was then pyrolyzed at 600 °C in OTF-1200X-8a tubular furnace (Kejing, Shenzhen, China) under a limited oxygen environment, with nitrogen serving as the inert gas. The resulting biochar was finally dried in a vacuum oven at 100 °C for 24 h.

Biochar (10 mg) was immersed in an excess amount of TTO/ethanol solution (100 mg/L) for 12 h to ensure near-saturation of the adsorption sites on the biochar [41]. The mixture was collected by vacuum filtration, dried, and weighed. The final product was designated as BC@TTO. The loading capacity (q, mg·g^−1^) of BC for TTO was calculated as Equation (1).

*q* = (*C*_0_ − *C*)*V*/*m* (1)

Here *C*_0_ and *C* are the initial and final concentrations of TTO (mg·L^−1^), *V* is the solution volume (L), and *m* is the mass of BC (g). TTO concentration was determined using a UV-2600 spectrophotometer (Shimadzu, Kyoto, Japan) at 273 nm, based on a pre-established standard curve of TTO in ethanol.

### 4.3. Adsorption Kinetics and Isotherm of BC for TTO

The adsorption behavior of BC for TTO was evaluated through isotherm and kinetic studies according to the procedure outlined in ASTM D3860-98 [42].

Adsorption isotherm: BC (10 mg) was introduced into 25 mL of TTO/ethanol solutions at varying initial concentrations (10, 20, 40, 60, 80, 100, 150, 200, 300 mg/L). The mixtures were incubated in the dark for 12 h to reach adsorption equilibrium. After filtration, the TTO concentration in the filtrate was determined by measuring absorbance at 273 nm using a UV-Vis spectrophotometer. The equilibrium adsorption capacity (*Q*_e_, mg/g) was calculated using Equation (2).

*Q*_e_(mg/g) = (*C*_0_ − *C*_e_) × *V*/m (2)

Here *C*_0_, *C*_e_, *V*, and *m* represent the initial concentration of TTO (mg/L), the final concentration of TTO (mg/L), solution volume (L), and mass of BC (g), respectively.

Adsorption kinetics: BC (10 mg) was mixed with 20 mL TTO/ethanol solution (20 mg/L). The mixture was kept in the dark and sampled at predetermined time intervals (10, 20, 30, 60, 90, 120, 240, 600 min). At each interval, the solution was filtered, and the residual TTO concentration was analyzed using a UV-Vis spectrophotometer. The adsorption capacity at time t (*Q*_t_, mg/g) was calculated using Equation (3).

*Q*_t_(mg/g) = (*C*_0_ − *C*_t_) × *V*/m (3)

Here *m*, *C*_0_, *C*_t_, and *V* represent the weight of BC (g), the initial concentration of TTO solution (mg/L), the concentration of TTO solution at time *t* (mg/L), and the volume of TTO solution (L), respectively.

### 4.4. Structural Characterization of BC and BC@TTO

Functional groups of lotus stalks and BC were identified FTS 3000 Fourier transform infrared spectroscopy (FTIR, Hercules, CA, USA). Spectra were acquired in transmission mode across the range of 400–4000 cm^−1^. Crystal structure of lotus stalks and BC was analyzed using an X’Pert Pro MPD diffractometer (XRD, Philips, Amsterdam, The Netherlands) with Cu Kα radiation. Scans were performed from 5° to 80° (2*θ*) at a scan rate of 10 °/min. Textural properties of BC, including specific surface area and pore size distribution, were determined using an ASAP 2020 HD88 analyzer (BET, Micromeritics, Norcross, GA, USA) via N_2_ physisorption at 120 °C. Surface morphology of BC were examined using a Sigma 300 field-emission scanning electron microscope (SEM, ZEISS, Oberkochen, Germany). Samples were sputter-coated with gold.

### 4.5. Preparation of Chitosan-Based Active Packaging Membranes

Chitosan-based active packaging membranes were fabricated using a solution casting technique. The membrane-forming solution consisted of CS (2 g), glycerol (0.6 g), and deionized water (70 mL) as the solvent. To ensure complete dissolution—by disrupting the hydrogen bonds within the CS structure—the mixture was heated to 95 °C and stirred continuously at 300 rpm for 60 min. At this pH (approximately 6–7), the CS dissolved thoroughly with no observable undissolved particles. For composite membranes, BC@TTO was incorporated at mass loadings of 1%, 3%, 5%, and 7% relative to chitosan. All components were combined, heated to 95 °C, and stirred continuously at 300 r/min for 60 min. The solution was then cast into a mold and dried in a vacuum oven at 65 °C for 12 h to produce the final membranes, which were labeled as CS, CS-1BC@TTO, CS-3BC@TTO, CS-5BC@TTO, and CS-7BC@TTO, respectively.

### 4.6. Structural Characterization of Chitosan-Based Active Packaging Membranes

Functional groups, crystal structure, and fracture appearance of chitosan-based active packaging membranes were analyzed by FTIR spectroscopy, XRD, and SEM, respectively. The test was performed as described in Section 4.4.

### 4.7. Performance Test of Chitosan-Based Active Packaging Membranes

The mechanical, physical, barrier, and functional properties of the membranes were evaluated. Tensile strength and elongation at break of chitosan-based active packaging membranes were determined according to ASTM D882 [43] using an YG 061-1500 electronic universal testing machine (Yuanmao, Yantai, China) with a 50 mm initial grip separation and a crosshead speed of 50 mm/min. Transparency of membranes was assessed by measuring the light transmittance of samples using a UV-Vis spectrophotometer across 200–800 nm. The test methods of moisture absorption rate (*M*_c_, %), water vapor permeability (*WVP*, g·mm·m^−2^·day^−1^), oxygen transmission rate (*OTR*, g·m^−2^·day^−1^), and antioxidant activity (*R*_DPPH_, %) of the membranes were according to our previous report [14]. Antimicrobial activity against Escherichia coli and Staphylococcus aureus was evaluated using the shake flask method (GB/T 20944.3–2025, ASTM E2149) [44]. Film samples were incubated in nutrient broth containing 1 × 10^5^–1 × 10^6^ CFU/mL bacteria at 37 °C for 2 h under shaking (150 rpm). Viable colonies were counted after serial dilution and plating, and the antibacterial rate was calculated relative to a blank control. The results are presented as averages, with standard deviations indicated.

### 4.8. Release Kinetics of TTO from Chitosan-Based Active Packaging Membranes

The release behavior of TTO from the composite membranes was quantitatively studied in vitro using a UV-Vis spectrophotometry. A standard calibration curve was established by plotting the absorbance of TTO/ethanol solutions at 273 nm against their known concentrations, providing a linear relationship for quantification. The membrane (0.2 g) was placed inside a pre-treated dialysis bag, and immersed in 100 mL of 5% (*v*/*v*) ethanol solution as the release medium. This concentration of ethanol was chosen because it provides a reasonable approximation of food-related environments, mimicking the polarity and solubility of matrices that may contain oils, water, or other volatile compounds, and is effective at solvating and releasing essential oils such as TTO. At predetermined time intervals, 3 mL of the medium was withdrawn and replaced with an equal volume of fresh medium to maintain sink conditions. The absorbance of the collected samples was measured at 273 nm, and the cumulative release of TTO was calculated based on the calibration curve. The release data were fitted to the Avrami model as shown in Equation (4) to analyze the release mechanism.

*R*(%) = 1 − exp(−(*kt*)*^n^*) (4)

Here *R*, *k*, and *n* represent the fractional release at time, the release rate constant, and the diffusional exponent indicative of the release mechanism, respectively.

### 4.9. Statistical Analysis

Results are reported as means ± standard deviations at least three times. Statistical analysis was performed using one-way analysis of variance (ANOVA) followed by appropriate Tukey’s test to determine significant differences among experimental groups. A *p*-value of less than 0.05 was considered statistically significant.

## 5. Conclusions

In this study, a novel active packaging membrane was successfully developed by integrating TTO-loaded lotus stalk biochar into a chitosan matrix. The biochar exhibited a mesoporous structure and high adsorption capacity for TTO, which was well described by pseudo-first-order kinetics and the Langmuir isotherm. The incorporation of BC@TTO into chitosan membranes improved their mechanical strength, barrier properties, and functional performance, including antioxidant and antibacterial activities. The optimal composite membrane (CS-3BC@TTO) demonstrated balanced properties, enhancing the preservation of blueberries by reducing weight loss during storage. The release of TTO from the membrane followed a diffusion-controlled mechanism, indicating potential for sustained active functionality. The composite membranes suffered from decreased transparency, which may limit visual appeal in certain packaging applications. Exploration of alternative biopolymer matrices or multilayer structures to improve optical clarity without compromising active properties.

## Data Availability

The relevant data pertaining to the findings of this study may be obtained from the corresponding authors upon request.

## Abbreviations

The following abbreviations are used in this manuscript:

BC	Biochar
TTO	Tea tree oil
CS	Chitosan
WVP	Water vapor permeability
OTR	Oxygen transmission rate
DPPH	2,2-Diphenyl-1-picrylhydrazyl
FTIR	Fourier transform infrared spectroscopy
XRD	X-ray diffraction
SEM	Scanning electron microscopy
BET	Brunauer–Emmett–Teller
